# Supplementary material for: Efficient Helium Separation with Two-Dimensional Metal–Organic Framework Fe/Ni-PTC: A Theoretical Study
Source: Membranes (Basel). 2021 Nov 26;11(12):927. doi: 10.3390/membranes11120927 (PMC8708020; doi:10.3390/membranes11120927)
Supplement: Supplementary file 1 [file membranes-11-00927-s001.zip › membranes-1466932-supplementary.pdf]

Supplementary Information for
Efficient helium separation with two-dimensional metal-organic framework: Fe/Ni-PTC

Jingyuan Wang [1], Yixiang Li [1], Yanmei Yang [2], Yong-Qiang Li [1], Mingwen Zhao [1], Weifeng Li [1], Jing Guan [1,*], Yuanyuan Qu [1,*]

[1] School of Physics, Shandong University, Jinan, 250100, Shandong, China; 1327942320@qq.com (J.W.); lyx1113@mail.sdu.edu.cn (Y.L.); yqli@sdu.edu.cn(Y.L.); zmw@sdu.edu.cn (M.Z.); lwf@sdu.edu.cn (W.L.); guanjing@sdu.edu.cn (J.G.); quyuanyuan@sdu.edu.cn (Y.Q.)

[2] College of Chemistry, Chemical Engineering and Materials Science, Collaborative Innovation Center of Functionalized Probes for Chemical Imaging in Universities of Shandong, Key Laboratory of Molecular and Nano Probes, Ministry of Education, Institute of Molecular and Nano Science, Shandong Normal University, Jinan, 250014, China; yym@sdnu.edu.cn

[*] Correspondence: quyuanyuan@sdu.edu.cn or guanjing@sdu.edu.cn

Supplementary Figures

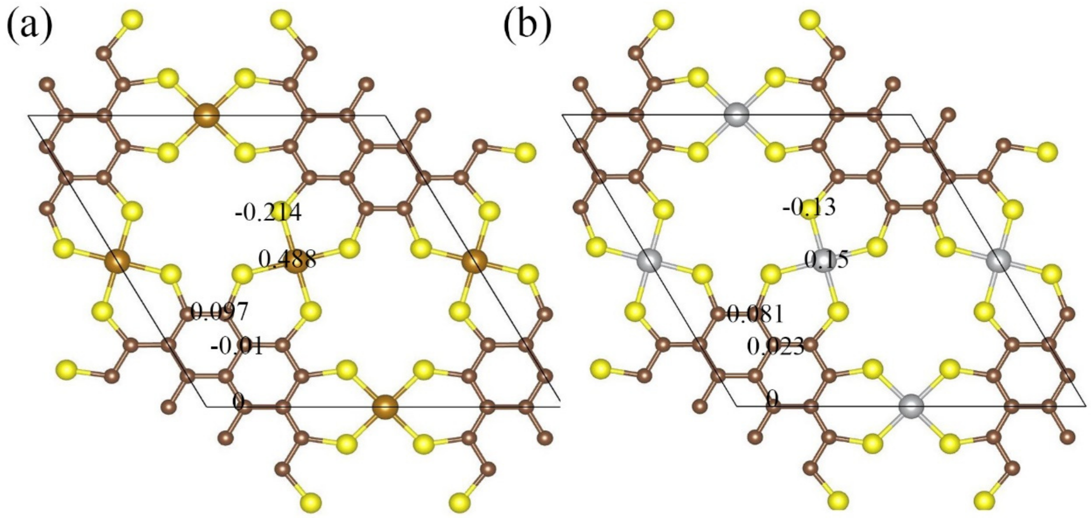

Figure S1. The atomic charges for (a) Fe-PTC membrane; (b) Ni-PTC membrane where the charges are denoted on respective atoms.

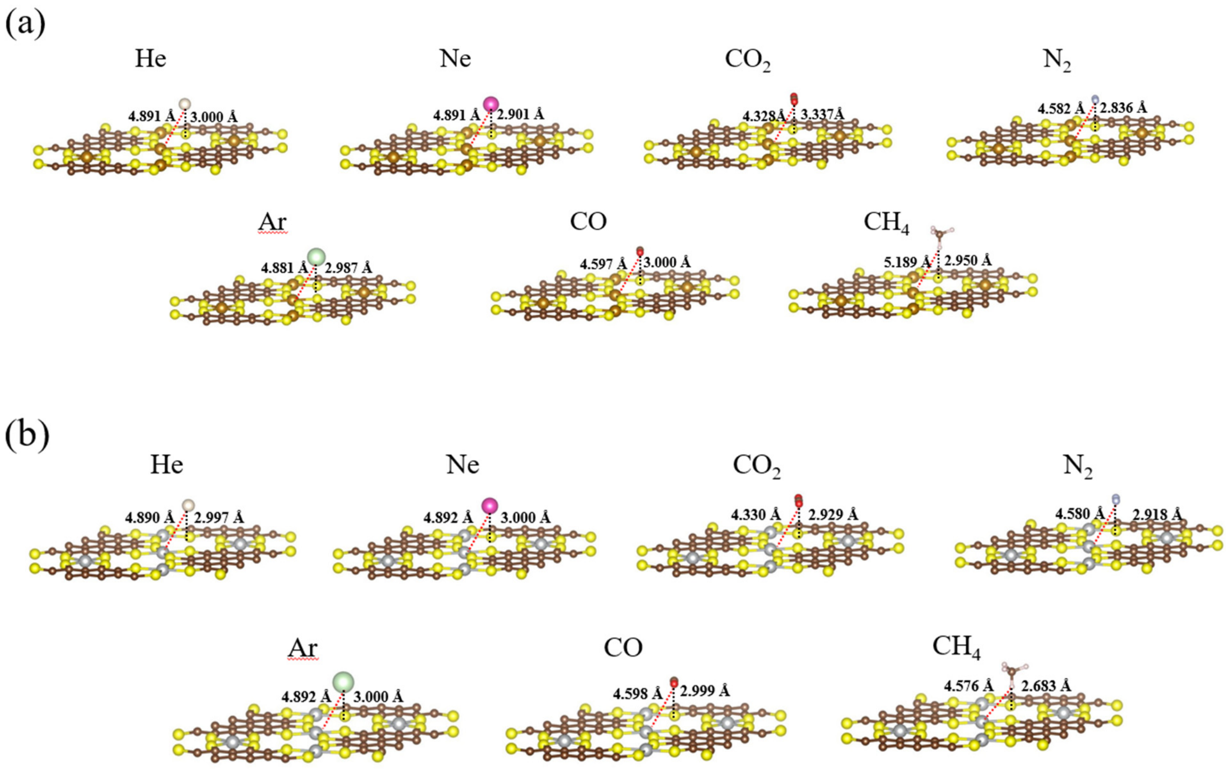

Figure S2. The schematic diagram of the adsorption state of each gas molecule on the (a)Fe-PTC and (b) Ni-PTC membranes. The black dotted lines represents the distance from the gas molecules to the membrane, and the red dotted lines represents the distance from the gas molecules to the Fe/Ni cation.

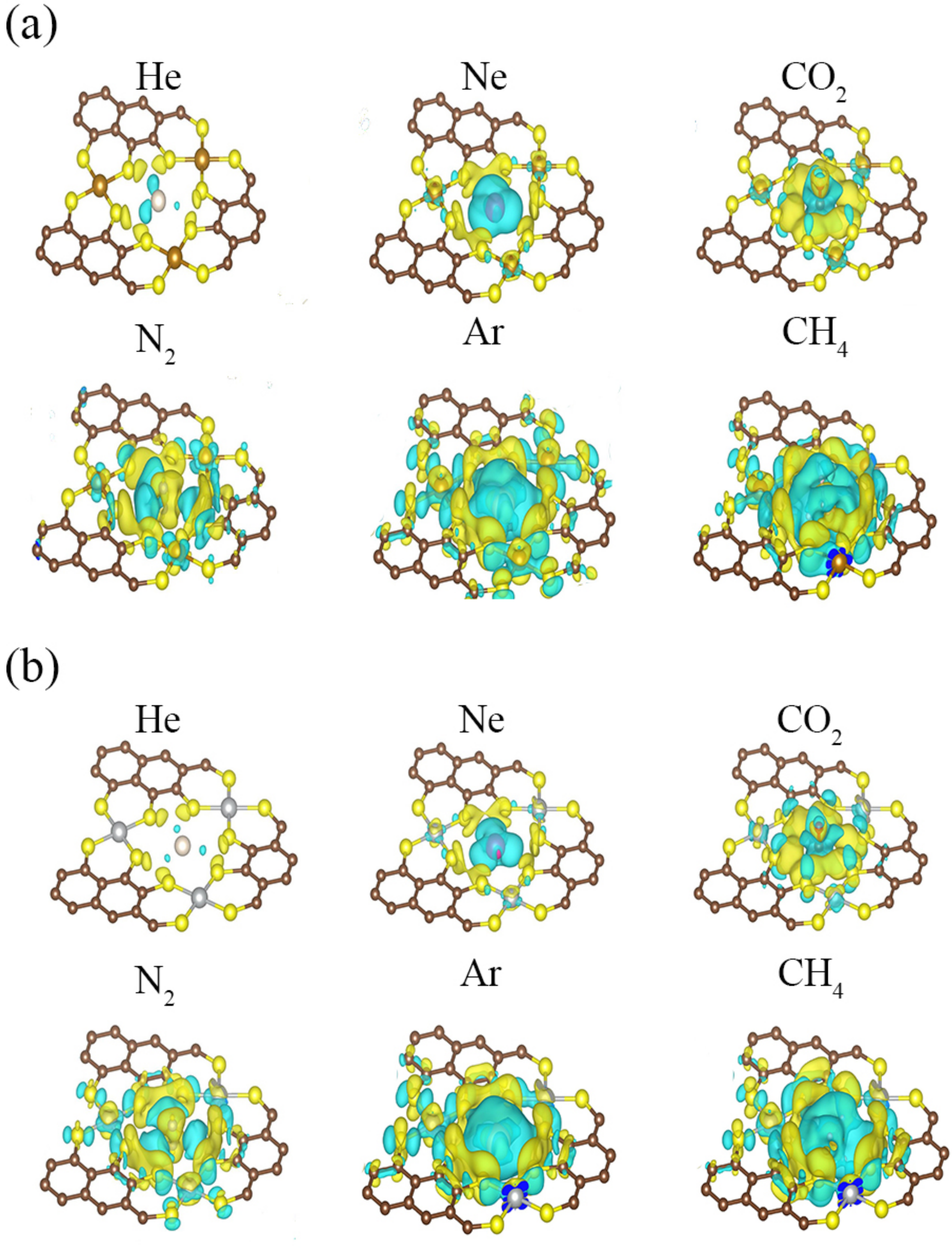

Figure S3. The eletron density difference maps at the transition state for both (a) gas-Fe-PTC and (b) gas-Ni-PTC systems. (isovalue of 0.0003Å^{-3})

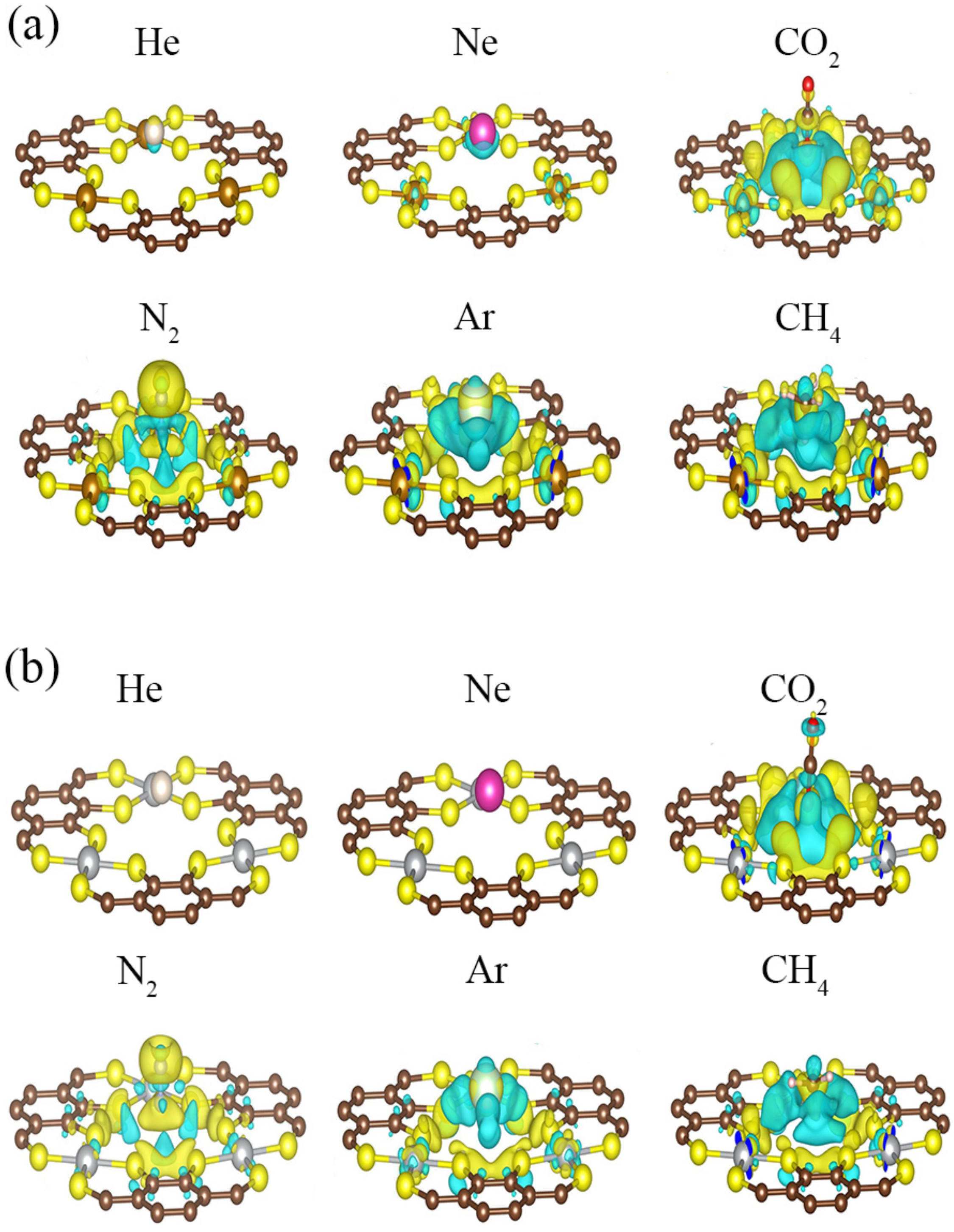

Figure S4. The eletron density difference maps at the state near the transition state for both (a) gas-Fe-PTC and (b) gas-Ni-PTC systems. (isovalue of 0.0003Å^{-3})

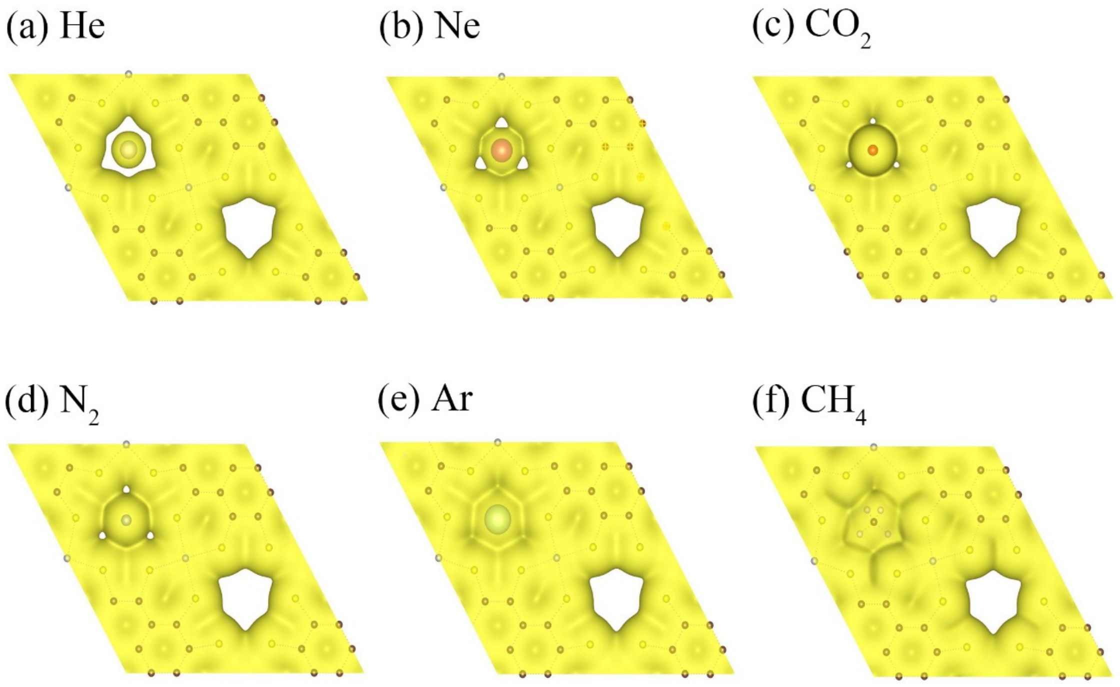

Figure S5. Electron-density isosurfaces for (a) He, (b) Ne, (c) CO_2, (d) N_2, (e) Ar, and (f) CH_4 molecules passing through the pore of the Ni-PTC membrane (isovalue of 0.01 Å^{-3}).

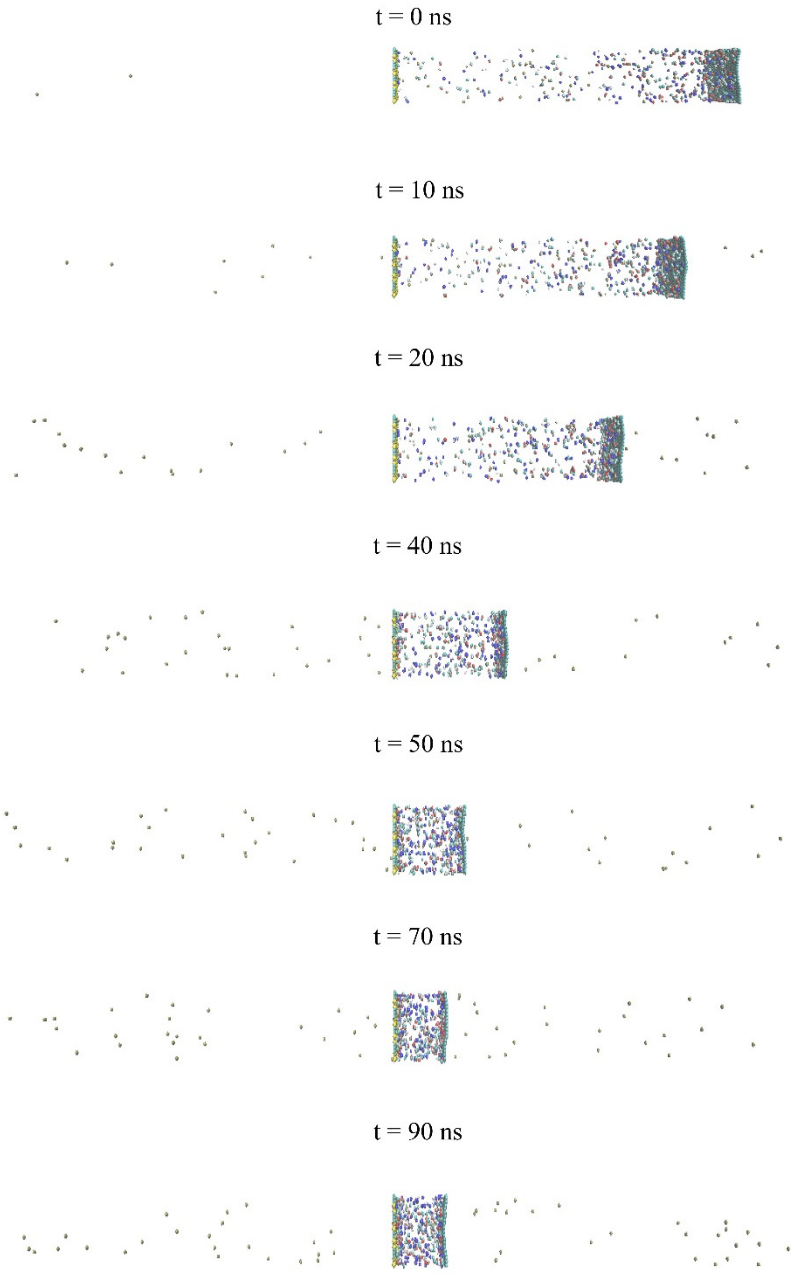

Figure S6. The snapshots of the gas mixture permeating through Fe-PTC membrane.

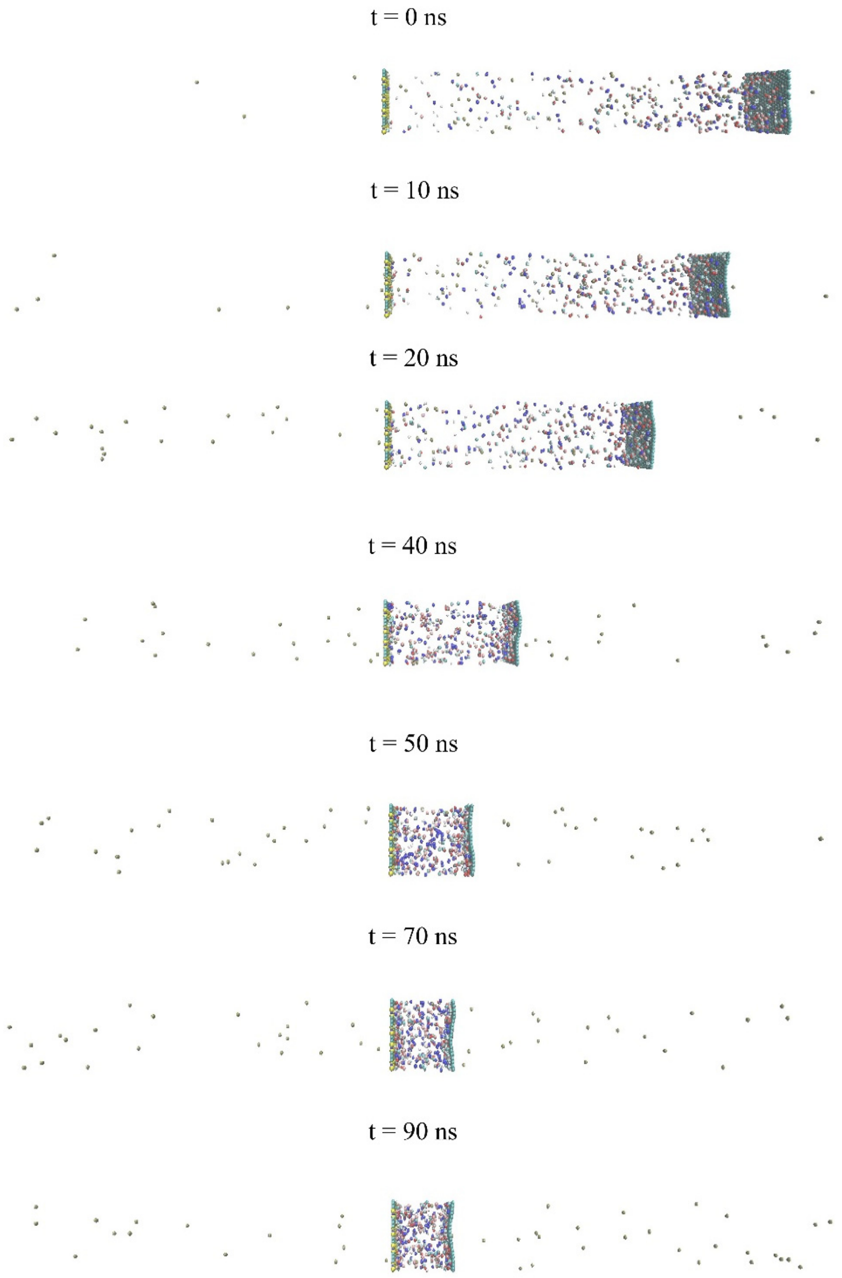

Figure S7. The snapshots of the gas mixture permeating through Ni-PTC membrane.

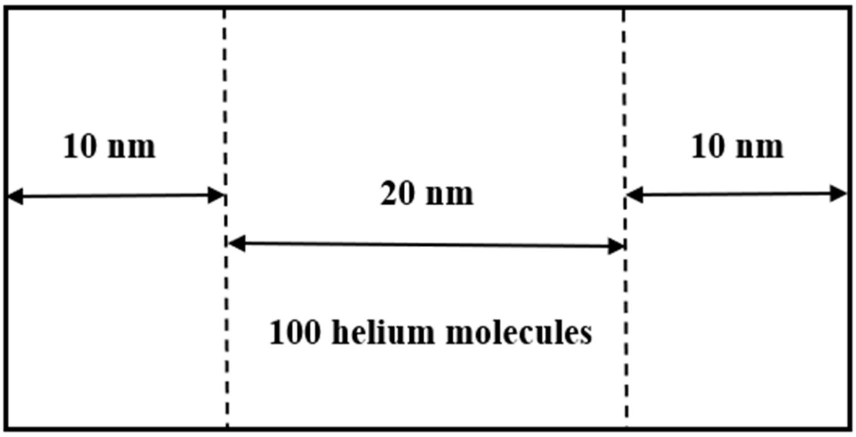

Figure S8. Molecular dynamics model for calculating permeance. The positions of Fe/Ni-PTC membranes are indicated by the dotted lines.

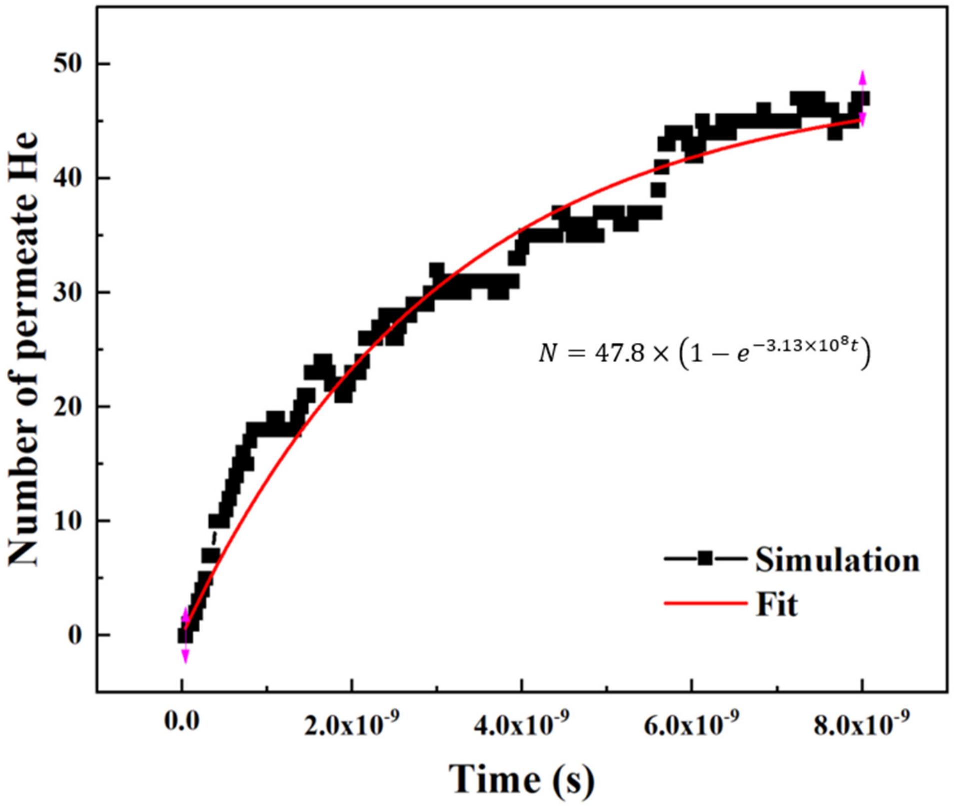

Figure S9. The number of permeated He molecules versus simulation time.

Supplementary Tables

Table S1. The convergence of the energy cutoff and the k-point mesh.

Membrane	K-points	Cutoff (eV)	System energy (eV)	Energy/atom (eV)
Fe-PTC	3 × 3 × 1	500	-281.0441	-7.2063
		550	-281.0529	-7.2065
	5 × 5 × 1	500	-281.0479	-7.2064
		550	-281.0565	-7.2066
	7 × 7 × 1	500	-281.0476	-7.2063
Ni-PTC	3 × 3 × 1	500	-281.8433	-7.2267
		550	-281.8509	-7.2269
	5 × 5 × 1	500	-281.8368	-7.2266
		550	-281.8433	-7.2268
	7 × 7 × 1	500	-281.8431	-7.2268

Table S2. The optimized vectors (a and b) upon gas adsorption, the vector changes percentage upon gas adsorption and the single point energy difference between complexes with unoptimized vectors and that with optimized vectors upon gas adsorption (ΔE).

Membrane	Gas	a = b (Å)	a (b) changes percentage	ΔE (eV)
Fe-PTC	He	13.55545	-0.034%	0.0005
	Ne	13.54668	-0.098%	0.0109
	CO_2	13.55272	-0.054%	0.0047
	N_2	13.55653	-0.026%	0.0003
	CO	13.54922	-0.079%	0.0049
	Ar	13.55370	-0.046%	0.0009
	CH_4	13.55078	-0.068%	0.0227
Ni-PTC	He	13.57258	+0.075%	0.0331
	Ne	13.56926	+0.068%	0.0135
	CO_2	13.56312	+0.005%	0.0009
	N_2	13.57570	+0.098%	0.0019
	CO	13.57236	+0.073%	0.0028
	Ar	13.57218	+0.072%	0.0024
	CH_4	13.57020	+0.058%	0.0008

Table S3. Force field parameters (van der Waals terms and partial charges) for CO_2 and N_2.

CO_2			
	ε (K)	σ (Å)	Q (e)
C	28.13	2.757	0.6512
O	80.51	3.033	-0.3256
bonds	length(Å)		
C-O	1.149		
N_2			
	ε (K)	σ (Å)	Q (e)
N	36.4	3.318	-0.4048
Center-Of-Mass	0	0	0.8096
bonds	length (Å)		
N-N	1.098		

Table S4. Atomic charges for CO and CH_4 molecules

CO		CH_4	
	Q (e)		Q (e)
C	0.0344	C	-0.3520
O	-0.0344	H	0.0880

Supplementary Method

Method for calculating permeance

In the simulation system to calculate He permeance, we randomly placed 100 helium molecules in between two layers of Fe/Ni-PTC membrane, while the x, y and z coordinates of the Fe/Ni-PTC membrane were kept frozen. The simulation box has a dimension of 4.068 nm × 4.698 nm × 40 nm. The molecular dynamics calculation model is shown in Figure S8. First, the permeance of helium through the membrane material was tested at 300 K. The relationship between flux J (mol s^{-1}) and permeance S (mol s^{-1} m^{-2} Pa^{-1}) is defined by the following formula [1-2]:

$$J = \frac{1}{N_A}\frac{dN}{dt} = A_g \cdot \Delta P \cdot S \qquad (1)$$

where A_g is the membrane area of Fe/Ni-PTC used in our MD simulations (A_g = 3.82 × 10^{-17} m^2), ΔP is the pressure drop across the membrane, N is the number of permeated He molecules, t is the MD simulation time, and N_A is the Avogadro constant. ΔP is dependent on the molecular numbers N. The initial pressure is estimated to be 9.16 × 10^5 Pa based on ideal gas law. Therefore, the expression for ΔP is as follows:

$$\Delta P = \frac{100 - N_{ad} - 2N}{100} \times 9.16 \times 10^5\ Pa \qquad (2)$$

where N_{ad} stands for the average number of He molecules adsorbed on the Fe/Ni-PTC membrane. After integrating Eq. (1), we obtained:

$$\mathrm{N} = 47.8 \times (1 - \mathrm{e}^{-4.98 \times 10^{11} \mathrm{St}}) \qquad (3)$$

in which B = 4.98 × 10^{11} S is the exponent of time decay. Then, Eq. (3) is applied to fit the time-dependent number profiles of permeated He molecules (Figure S9). The fitted parameter B = 4.98 × 10^{11} S is calculated to be 3.13/3.29 × 10^8, and thus the He permeance (S) is found to be 6.3/6.6 × 10^{-4} mol s^{-1} m^{-2} Pa^{-1}. Based on the same method, the He permeances of Fe/Ni-PTC membrane at different temperatures have also been computed.
